# Piezocatalytic techniques and materials for degradation of organic pollutants from aqueous solution

**DOI:** 10.1016/j.eehl.2024.08.001

**Published:** 2024-08-22

**Authors:** Bo Liu, Xiaolu Liu, Yang Li, Muliang Xiao, Zhongshan Chen, Suhua Wang, Hongqing Wang, Xiangke Wang

**Affiliations:** a School of Chemistry and Chemical Engineering, University of South China, Hengyang 421001, China; b College of Environmental Science and Engineering, North China Electric Power University, Beijing 102206, China; c School of Environmental Science and Engineering, Guangdong University of Petrochemical Technology, Maoming 525000, China

**Keywords:** Organic pollutants, Piezoelectric techniques, Advanced oxidation process, Piezoelectric material

## Abstract

With the rapid development of industry, agriculture, and urbanization, various organic pollutants have accumulated in natural water, posing a potential threat to both the ecological environment and human beings, and removing organic pollutants from water is an urgent priority. Piezoelectric techniques, with the advantages of green, simple operation, and high efficiency, are highly sought after in the degradation of environmental organic pollutants. Moreover, combining piezoelectric techniques with advanced oxidation processes (AOPs), photocatalysis, or electrocatalysis can further effectively promote the efficient degradation of target pollutants. Therefore, a perspective is presented on the recent progress of piezoelectric techniques for the degradation of various organic pollutants from aqueous solutions. The classification of various piezoelectric materials, as well as modification strategies for improving piezocatalysis, are first systematically summarized. Furthermore, the latest research on piezocatalysis and its combination with other technologies, such as AOPs, photocatalysis, and electrocatalysis, in the degradation of environmental pollutants is discussed. The potential mechanisms of piezocatalysis are also analyzed in depth. Finally, the urgent challenges and future opportunities for piezoelectric techniques in the degradation of organic pollutants are provided.

## Highlights

• Piezocatalytic techniques in organic pollutants degradation were summarized.
• Different piezocatalytic materials were described.
• Future applications of piezocatalytic techniques in environmental pollution management were anticipated.

## Graphical abstract

**Figure:**
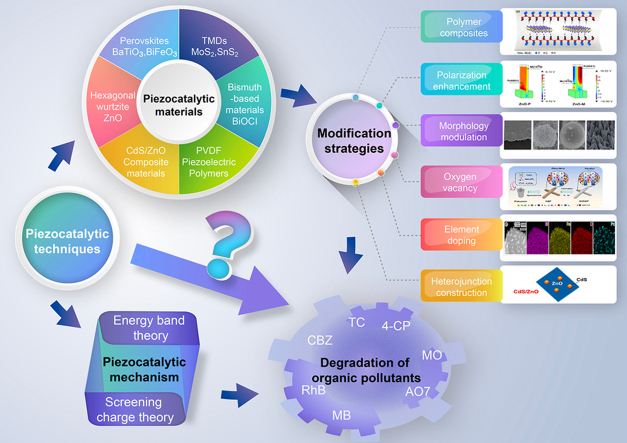

## 1 Introduction

Water is a precious resource, serving as an essential foundation for the entire earth's ecosystem and human society [[1], [2], [3], [4]]. Nevertheless, with the rapid development of industry, agriculture, and urbanization, significant quantities of wastewater and domestic sewage flow into natural water, exerting serious threats to the ecosystem and human health [5,6]. In particular, the organic pollutants, such as dyes (acid orange [AO7], methylene blue [MB], rhodamine B [RhB] and methyl orange [MO]), antibiotics (4-chlorophenol [4-CP], carbamazepine [CBZ], tetracycline [TC]), and Bisphenol A (BPA), in contaminated water may accumulate in the human body through the food chain, leading to irreversible damage to the liver, reproductive system, ecosystem, and other vital aspects [7,8]. Therefore, developing efficient technologies to remove organic pollutants from polluted water bodies is a significant issue that must be addressed.

To date, despite various methods such as physical methods, chemical methods, and biological methods, eliminating organic pollutants from contaminated water remains a challenge [[9], [10], [11]]. Physical methods, such as adsorption, are easy to implement, but they essentially transfer pollutants from one substance to another and easily lead to re-contamination [12]. Chemical methods such as ozonation and chlorination can thoroughly eliminate pollutants, but they are prone to generating toxic by-products that cause secondary pollutants [13,14]. Although biological techniques are cost-effective and eco-friendly, they are limited by the long reaction times and potential reversibility [15]. Recently, photocatalysis, or electrocatalysis combined with advanced oxidation processes (AOPs), has been studied extensively [16]. However, the photocatalytic efficiency is affected by the narrow light absorption range and the fast recombination of photogenerated carriers, while electrochemical methods are limited by the high cost and energy consumption. Therefore, it is desirable to develop a technology that is eco-friendly, efficient, and low in energy consumption.

Piezocatalysis, an emerging green chemistry technology that can utilize mechanical energy, has attracted significant attention [17]. Under the excitation of external mechanical forces, some materials deform and spontaneously polarize to induce an internal electric field/piezopotential, facilitating charge carriers separation and transfer to trigger the redox reaction, piezocatalysis [18]. These materials that can produce a piezoelectric effect by generating charges in response to external mechanical forces are called piezoelectric materials. Using piezoelectric techniques, organic pollutants can be degraded in a very green, simple, and efficient way. For example, Bi_2_WO_6_ nanosheets exhibited high piezocatalytic performance for the removal of RhB [19] and MO [20]. Ba_0.75_Sr_0.25_TiO_3_ nanoparticles could decompose dyes by collecting mechanical energy from the environment through friction, and 99.0% RhB was degraded under the mild stirring of 300 rpm within 3 h [21].

Piezocatalytic techniques enable the harvest of external mechanical energy, or even small, unused water flow, tide, wind power, and vibration caused by human activities, offering a novel approach for repurposing waste mechanical energy [[22], [23], [24]]. Compared to traditional electrocatalysis, piezoelectric catalysis has the advantage of not requiring additional electrical energy. Moreover, the internal electric field generated by the piezoelectric material can effectively inhibit the photogenerated carriers' recombination, contributing to excellent catalytic performance. As a result, a lot of researchers have turned their attention to catalysis augmented by the piezoelectric effect in the application of degradation of organic pollutants, such as piezocatalysis in combination with photocatalysis, electrocatalysis or AOPs [[24], [25], [26]].

Up to now, some reviews about piezocatalysis have been reported. Wang et al. [27] summarized the progress of piezocatalytic material design strategies. Zheng et al. [28] summarized the recent progress of piezoelectric materials in piezocatalysis and piezo-photocatalysis. Liu et al. [29] reported the application of piezocatalytic techniques for environmental remediation. However, a comprehensive overview of piezoelectric materials, piezocatalytic techniques, and their combination with photocatalysis, electrocatalysis, or AOPs for the degradation of organic pollutants is still rare. From this perspective, the use of piezoelectric techniques for the degradation of organic pollutants from water systems is comprehensively summarized (Fig. S1). The classification and modification strategies of piezoelectric materials were first introduced. Next, the latest research progress on the degradation of organic pollutants from aqueous solutions using piezoelectric techniques was discussed. The mechanism of the piezoelectric techniques in the degradation of environmental pollutants was discussed in detail. The existing challenges and future opportunities for piezoelectric techniques in environmental remediation are then proposed.

## 2 Piezocatalytic techniques for degradation of organic pollutants

Organic pollutants are seriously harmful to human health and the natural ecological environment. As an emerging green chemistry technology, piezocatalysis can use renewable mechanical energy from nature and convert it into chemical energy. Given the fascinating advantages of low cost, no pollution, mild reaction conditions, and light independence, piezocatalysis shows considerable potential in the remediation of environmental organic pollution.

### 2.1 Classification and modification strategies of piezoelectric materials

#### 2.1.1 Classification of piezoelectric materials

Piezoelectric materials can be generally divided into four categories: piezoelectric single crystal, piezoelectric ceramics, organic piezoelectric materials (piezoelectric polymers), and composite piezoelectric materials composed of them. The performance of piezoelectric single crystals is relatively stable [30]. Piezoelectric ceramics are a class of ceramic materials with a piezoelectric effect after oxides are mixed and sintered at high temperatures [31]. Piezoelectric polymers generally have inferior piezoelectric responses but better flexibility than piezoelectric single crystals and ceramics [32]. When a single piezoelectric material cannot meet the requirements, composite piezoelectric materials can be customized by selecting and combining multiple materials to attain the desired properties.

#### 2.1.2 Modification strategies for improving piezocatalysis

Although some progress has been made in the design of piezoelectric materials, there are still some drawbacks, such as bad intrinsic piezoelectric responses of most piezoelectric materials, absence of active sites, and low utilization rate of piezoelectric charges [33]. So, how can more efficient piezocatalysis be achieved?

The piezoelectric coefficient *d*_33_ is the primary parameter used to measure the conversion efficiency between mechanical energy and electricity of piezoelectric materials, which can be increased by changing the crystal structure, crystal orientation, and chemical composition of the material. A higher piezoelectric coefficient will generally lead to better performance [34]. Additionally, during the photocatalytic reaction process, charge carriers are in dynamic equilibrium between separation and recombination, and the migrating charge carriers may recombine and dissipate the energy as heat. The piezocatalytic reaction can be triggered only when the charge carriers successfully migrate to surface reactive sites [35]. Efficient piezoelectric materials usually feature a large surface area, abundant active sites, suitable band positions, and improved charge separation. Given these, a variety of modification strategies, including morphology modulation, oxygen vacancy (OV), elemental doping, polarization enhancement, heterojunction construction, and polymer composites, can be used to attain highly efficient piezocatalytic performance. The advantages and disadvantages of different modification strategies are summarized in Table S1.

#### 2.1.3 Common high-efficiency piezocatalytic materials

Recently, many piezocatalytic materials, such as hexagonal wurtzite ZnO [36], perovskites (e.g., BaTiO_3_ [37], BiFeO_3_ [38]), transition metal disulfide compounds (TMDs) (e.g., MoS_2_ [39], SnS_2_ [40]), layered bismuth-based materials (e.g., BiOCl [41]) and composite materials (e.g., CdS/ZnO [42], BaTiO_3_/PVDF [43]), have been reported. ZnO is a piezoelectric single crystal with strong stability. Perovskites, a piezoelectric ceramic, are the most widely used piezoelectric material because of their excellent piezoelectric properties [44]. Transition metal disulfide compounds are the typical 2D piezoelectric materials [45,46]. Layered bismuth-based compounds, such as BiOIO_3_, BiOClO_3_, Bi_5_O_7_I, Bi_12_O_15_C_l6_, and BiOX (X = Cl, Br, I), demonstrate exceptional piezoelectric properties attributable to their unique layered structures [47,48]. Polyvinylidene fluoride (PVDF), as a piezoelectric polymer, is favorable to the synthesis of composite piezoelectric materials owing to its recyclability, strong flexibility, and easy preparation [49]. Other piezoelectric materials, including h-BN, carbon nitride, and emerging metal–organic frameworks (MOFs), have also attracted much attention [46,50,51].

### 2.2 Piezocatalytic techniques for degradation of organic pollutants

Piezocatalysis can degrade pollutants into non-toxic substances or valuable products through mechanical energy [52]. Piezoelectric materials exhibit the piezoelectric effect, whereby, under the influence of external pressure or electric field, charge separation occurs within the material. This results in an asymmetric distribution of positive and negative charges in the material, ultimately leading to the generation of a voltage or electric field. With the characteristic of allowing the conversion between mechanical energy and electrical energy, piezoelectric materials are widely applied in environmental pollution management and other areas [[53], [54], [55]]. The progress of piezocatalysis and its combination with other techniques in the degradation of organic pollutants is mainly discussed below, and the properties of some piezocatalytic materials related to the degradation of organic pollutants are summarized in Table 1.

**Table 1:** Comparison of degradation properties for organic pollutants by different piezocatalytic materials.

Piezocatalyst	Pollutants	Pollutants concentration	Catalytic condition	Degradation efficiency	Ref.
BaTiO_3_ microdendrites	AO7	10^−6^–10^−4^ mol/L	Ultrasonic vibration 25 °C	80%, 90 min	[62]
Bi_2_WO_6_ nanosheets	MB	10 mol/L	Ultrasonic vibration (40 kHz, 150 W)	∼96%, 35 min	[20]
ZnSnO_3_ nanoparticles	RhB	4.7 × 10^−6^ M	Ultrasonic vibration (33 ± 3 kHz, 120 W)	99.9%, 60 min	[85]
MoS_2_ nanoflowers	RhB	10 ppm	Ultrasonic wave (40 kHz, 250 W)	93%, 60 s	[86]
MoS_2_/carbon fiber	RhB	10 ppm	Wastewater flow	99.9%, 40 min	[87]
BaTiO_3_/C nanocomposites	RhB	5 mg/L	Ultrasonic vibration (40 kHz, 150 W)	75.5%, 40 min	[88]
Bi_1/2_Na_1/2_TiO_3_-based nanofibers	AO7 MO	5 mg/L 10 mg/L	Ultrasonic vibration (40 kHz, 100 W)	99.8% 95%	[89]
(Ba, Sr)TiO_3_	RhB	5 mg/L	Stirring, 300 rpm	99%, 3 h	[21]
tetragonal BaTiO_3_ particles	4-CP	25 mg/L	Ultrasonic vibration (40 kHz, 110 W)	71.1%, 120 min	[90]
Ba_0.5_Sr_0.5_TiO_3_	CBZ	10 mg/L	Ultrasonic vibration (40 kHz, 100 W)	94.5%, 30 min	[69]
Bi/BiOCl	CBZ	5 mg/L	Ultrasonic vibration (40 kHz, 150 W)	75.11%, 30 min	[41]
MoS_2_	TC RhB	10^−5^ mol/L 10 mg/L	Ultrasonic vibration (40 kHz, 110 W)	93%, 120 min 96%, 60 min	[91]
WS_2_	TC RhB	10^−5^ mol/L 10 mg/L	Ultrasonic vibration (40 kHz, 110 W)	71%, 120 min 65.7%, 60 min	
WSe_2_	TC RhB	10^−5^ mol/L 10 mg/L	Ultrasonic vibration (40 kHz, 110 W)	62%, 120 min 43.5%, 60 min	
Cu_3_B_2_O_6_	RhB	10 mg/L	Ball milling, 600 rpm	99.9%, 30 min	[92]
BiFeO_3_@CdS nanofibers	BPA	10 mg/L	Ultrasonic vibration (40 kHz, 300 W)	99.7%, 60 min	[93]

#### 2.2.1 Piezocatalysis for degradation of organic pollutants

ZnO, renowned for its straightforward synthesis process, eco-friendliness, and remarkable stability, stands out as one of the most extensively employed piezoelectric substances [56]. Up to now, various ZnO materials, such as ZnO nanorods/nanowires, ZnO nanoparticles, and ZnO nanosheets, have been used to degrade organic pollutants [[57], [58], [59], [60], [61]].

Perovskites also play a key role in piezocatalysis for the degradation of environmental organic pollutants. For example, Hong et al. [62] reported the application of BaTiO_3_ perovskite in the piezocatalytic degradation of organic pollutants. Under ultrasonic action, the charges in BaTiO_3_ microcrystals are induced and accumulated on the surface, forming a piezopotential, which enables the degradation of AO7. Other piezoelectric perovskite materials including Bi_4_Ti_3_O_12_ [63], (Ba, Sr)TiO_3_ [64], PbZr_x_Ti_1-x_O_3_ [65], SrTiO_3_ [44], K_0.5_Na_0.5_NbO_3_ [66], LiNbO_3_ [67], and NaNbO_3_ [63] have also been studied in piezoelectric organic degradation. Introducing substitutes into the perovskite lattice is an effective strategy to improve pristine piezoelectric properties. The existence of a substitute can lead to lattice asymmetry and obvious structure distortions, thereby leading to lattice mismatch, which is beneficial for improving piezocatalytic performance [68]. Moreover, the substituting ions occupy off-center positions to generate dipole moments, enhancing polarization behavior during the piezoelectric process [69]. Yu et al. [69] successfully synthesized Ba-substituted SrTiO_3_ materials and applied them to degrade CBZ from an aqueous solution. They found Ba_0.5_Sr_0.5_TiO_3_ (BSTO-2) exhibited the best degradation efficiency, achieving 94.5% degradation efficiency for CBZ (10 mg/L) in 30 min. The kinetic rate constant of BSTO-2 for CBZ degradation was 0.106 min^−1^, which was 1.86 times that of SrTiO_3_ and 2.08 times that of BaTiO_3_. The enhanced performance was attributed to an internal distorted structure and enhanced electronic transmission capability, leading to a higher piezoelectric response and a faster interfacial charge transfer rate.

Bismuth-based piezoelectric materials have also become a research hotspot in environmental pollution management. Zhu and co-workers [48] prepared Au-decorated bismuth oxybromide (Au–BiOBr) piezoelectric materials. They found that the introduction of Au nanoparticles accelerated charge transfer, enhanced light absorption, enlarged the internal electric field, and adjusted the arrangement of the band structure of BiOBr, thereby enhancing the piezoelectric performance. The CBZ removal efficiency of as-prepared Au–BiOBr reached 95.8% within 30 min [48].

MoS_2_, a two-dimensional piezoelectric material, is of great interest as a piezoelectric material in the field of piezoelectric degradation of organic pollutants owing to its distinguished piezoelectric response, excellent electronic properties, and exceptional mechanical flexibility. Lan et al. [70] developed a novel spiral reactor that used MoS_2_ sheets as piezocatalytic materials and utilized the movement of water as an external force-triggered piezocatalysis treatment of organic wastewater to enable a self-powered reaction and independent operation. The degradation performance of MoS_2_ for benzothiazole was up to 94.8% in 24 cycles of experiments, which was 8.8 and 4.9 times higher than that of the quiescent solution of MoS_2_ and commercial MoS_2_, respectively. Further experiments showed that this system showed excellent degradation performance in the degradation of multiple pollutants such as enrofloxacin, metronidazole, and ciprofloxacin. In this process, the moving water in the spiral reactor drove MoS_2_ to produce piezo-charges, which reacted with dissolved oxygen and water to generate reactive oxygen species (ROS) to achieve the purpose of degrading target organic pollutants. They found that the free radicals produced by the spiral reactor also had remarkable sterilization effects [71].

#### 2.2.2 Piezocatalytic techniques in combination with other techniques

Piezocatalytic techniques and their combination with AOPs, photocatalysis, or electrocatalysis have been widely applied to the field of degradation of environmental pollutants, and many piezoelectric materials have shown outstanding catalytic performance, such as ZnO, perovskites, bismuth-based material and molybdenum disulfide [25,26].

To enhance the separation performance of piezo-polarization-generated charges on the surface of ZnO, Zhu's group [56] synthesized hierarchical structures ZnO with abundant oxygen vacancies (OVs) (TB-ZnO), which activates peroxymonosulfate (PMS) under piezoelectric activation and realizes efficient degradation of ibuprofen. Theoretical calculations showed that the ZnO with medium OVs concentration achieved increased electron delocalization, decreased charge transfer barriers, and increased reactant affinity, thus accelerating the activation kinetics of PMS, which further demonstrated the quantitative relationship between OVs concentration and piezoelectric efficiency in ZnO [72].

The bismuth-based piezoelectric catalyst can also be used as the activator of peroxymonosulfate (PMS) to remove organic pollution by combining the piezoelectric technique and the AOPs system. Bismuth oxychloride nanosheets (BiOCl) piezocatalysts were further synthesized, and BiOCl was used as PMS activation under ultrasonic vibration to investigate the performance of CBZ removal [47]. Experiments exhibited that the removal efficiency of BiOCl piezo-activated PMS system for CBZ within 40 min reached 92.5%, which was 1.63 times that of BiOCl piezoelectric catalysis. The mechanism study showed that the piezoelectric field of BiOCl can enhance the adsorption of PMS and accelerate the production of active free radicals sulfate radical (•SO_4_^−^), hydroxyl radical (•OH), superoxide radical (•O_2_^−^) and singlet oxygen (^1^O_2_), resulting in the efficient degradation of CBZ from aqueous solution [47].

Xing's group [73] synthesized a Co_3_S_4_/MoS_2_ composite piezoelectric catalyst applied to the coupled piezoelectric technique and AOP system, realizing the conversion of organic pollutants into value-added products instead of CO_2_ for the first time. The organics were first oxidized to carbonate via PMS-based AOPs, and the carbonate was subsequently converted into CO by piezoelectric reduction in ultrasonic conditions using Co_3_S_4_/MoS_2_. Theoretical calculation showed that the introduction of Co_3_S_4_ could significantly promote the transfer and utilization of piezoelectric electrons and enhance the selective conversion of carbonate to CO.

The decomposition rate of MB of α-Fe_2_O_3_/PVDF composite under piezo-photocatalysis was 4.7 times higher than that under piezocatalysis, and the non-ultrasonic photocatalysis mediated by water flow also shows a high degradation efficiency of 83.9% within 180 min. The piezo-potential driven by ultrasound and water flow promoted the separation of photogenerated carriers, thereby increasing the formation of free radicals involved in the catalytic oxidation of pollutants [74].

In summary, as a green organic pollution treatment technology, piezoelectric catalysis has received more and more attention. The integration of piezocatalysis and other catalytic systems can make full use of various natural energy sources, such as wind and solar energy, to efficiently remove pollutants [75]. Combining piezoelectric technology with AOPs, photocatalysis, or electrocatalysis not only broadens the application range of piezoelectric technology but also overcomes the shortcomings of a single technology. All of these make piezoelectric catalysis one of the most promising technologies in water purification.

## 3 Mechanism of piezocatalysis for degradation of organic pollutants

At present, there are two popular piezocatalytic mechanisms: energy band theory and screening charge theory. According to the energy band theory, whether a piezocatalytic reaction can occur mainly depends on the energy band levels (conduction band [CB] and valence band [VB]) of the piezoelectric material. The piezopotential can regulate the energy band structure and induce charge carriers' separation and migration to the catalyst surface to trigger a reaction [76]. In terms of the screening charge theory, the piezopotential should first meet the requirement of the critical Gibbs free energy threshold for the redox reaction thermodynamically, which means the piezopotential should fully reach or exceed the required potential of the desired reaction [77]. The polarization change caused by the piezoelectric effect can promote the dynamic screening phenomenon. Then, under the influence of piezopotential, the screening charges over the surface of piezoelectric material adsorbed from the external system take part in the redox reaction.

It is widely accepted that the internal electric field or piezo-potential resulting from the piezoelectric effect is the driving force of the piezocatalytic reaction. Polarization promotes the migration of negative and positive charges in opposite directions, and the accumulation of dipole moments in the same direction forms an internal electric field or piezopotential. Under the influence of an internal electric field or piezo-potential, the free charge carriers (e^−^ and h^+^) migrate to the surface of piezoelectric material to trigger the piezo-catalytic reaction to degrade organic pollutants [[78], [79], [80], [81], [82], [83], [84]]. Generally, successful migration of charge carries to active sites on the surface of piezocatalyst by sufficient driving forces is necessary to ensure the occurrence of piezocatalytic reaction. Fig. S2 explains the detailed charge transfer mechanism in piezoelectric catalysis.

**Fig. 1:**
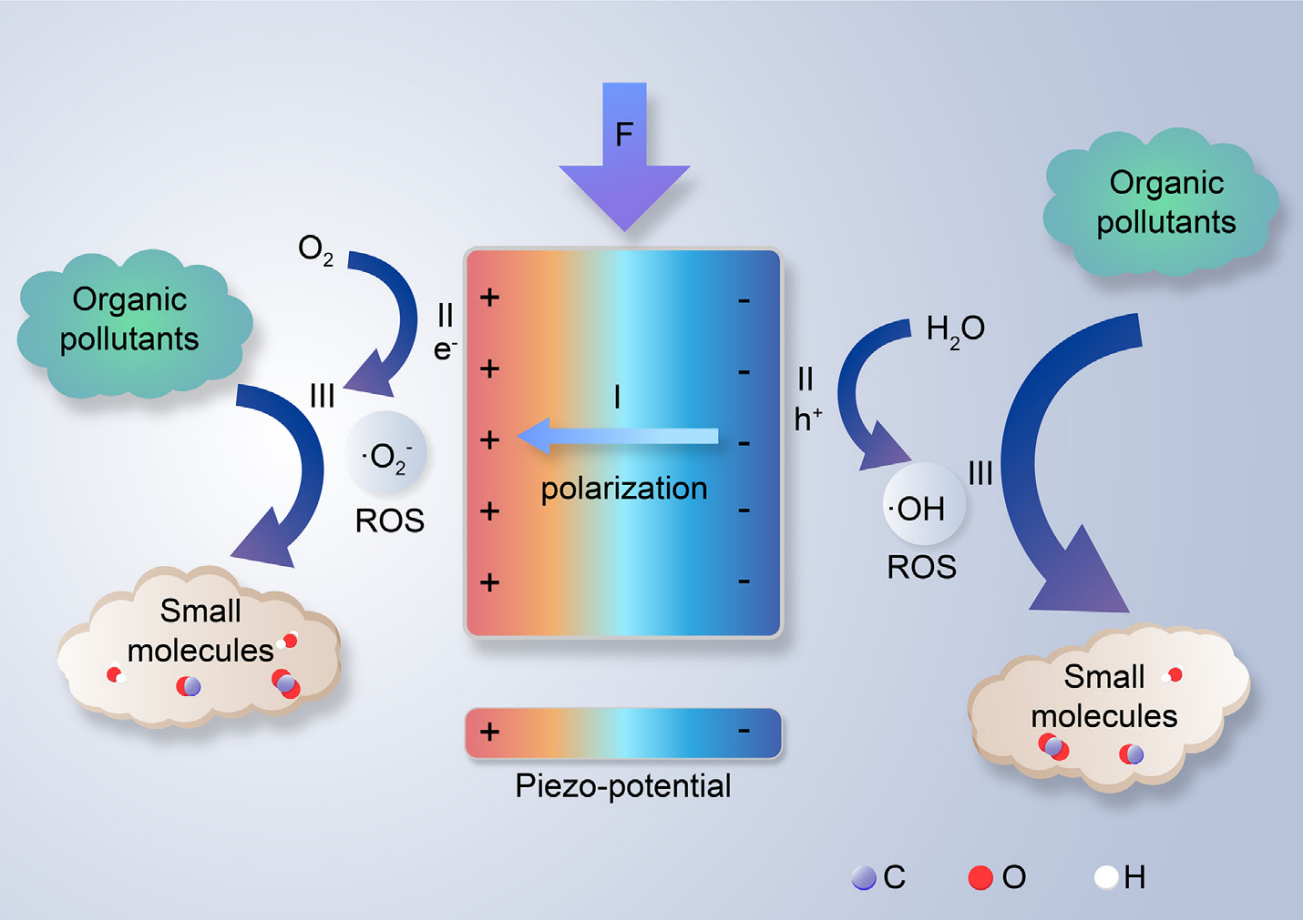
The schematic diagram of the reaction mechanisms for the piezoelectric degradation of organic pollutants. I) Piezoelectric effect: under the external mechanical forces, piezoelectric material deforms and spontaneously polarizes to induce a piezo-potential across it, then e^−^ and h^+^ are separated and transferred to the surface in opposite directions of piezoelectric material. II) Generation of ROS: e^–^ reacts with dissolved O_2_ to produce •O_2_^−^ in the negative charge regions, and •OH is generated by the reaction of holes and OH^−^ in the opposite area of the surface. III) Degradation of organic pollutants: the degradation of organic pollutants is achieved through a piezocatalytic reaction with ROS.

As shown in Fig. 1, the piezoelectric degradation of organic pollutants can typically be divided into the following three processes:

I) Piezoelectric effect:

Piezoelectric material → Piezoelectric material (e^−^ + h^+^)

II) Generation of ROS:

$$e^{-}+{\;O}_{2}\;\to \;•O_{2}^{-}$$

$$h^{+}+{\;H}_{2}O\;\to \;•\text{OH}$$

III) Degradation of organic pollutants:

ROS + organic pollutants → small molecules

The combination of piezocatalysis with other technologies, including AOPs, photocatalysis, and electrocatalysis, is a good way to achieve the goal of efficient degradation of organic pollutants. Take piezoelectric photocatalysis as an example. In the process of piezo-photo catalysis, the internal electric field formed by piezoelectric catalysis can accelerate the separation of the photogenerated carrier and prolong its lifetime. Moreover, the piezoelectric effect can cause tilting of the VB and CB of photocatalysts. Therefore, the photocatalytic capability of piezoelectric materials has been significantly enhanced. Furthermore, the adsorbed ions or charge carriers generated by photocatalysis may saturate the static piezoelectric potential, thereby reducing the driving force for charge separation. Consequently, piezo-photocatalysis requires periodic application of external force to repeat this process [25].

## 4 Conclusions and perspectives

In this perspective, the latest progress of piezoelectric techniques in the removal of environmental organics is presented. The classification of various piezoelectric materials is first introduced, and the modification strategies for improving piezocatalysis are discussed. Moreover, the piezoelectric technique or its combination with other technologies (AOPs, photocatalysis, electrocatalysis) in the degradation of environmental pollutants is comprehensively summarized.

To date, many piezoelectric materials have shown distinguished performance in the degradation of organic pollutants, but there are still some unresolved problems in the development of piezoelectric techniques.

(I) Their performance has remained less than satisfactory, so some methods should be taken to modify the internal structure of piezoelectric materials to promote the catalytic performance of piezoelectric materials, such as defect engineering, element doping, shape control, and heterojunction engineering.

(II) Currently, the main mechanical driving force of piezoelectric catalysis is ultrasound, which may damage the internal structure of piezoelectric materials and reduce the degradation effect of pollutants. Natural forces such as wind and water flow, with lower frequency, are gentle to the catalysts and eco-friendly for the environment. If a piezoelectric material is fixed in the ocean or river and replaced regularly, the degradation of pollutants in the natural water system can be easily achieved. To ensure that the desired goal is achieved, the following conditions must be met: i) The material can be recycled and does not pollute the environment, and polymer composite film should be a good choice; ii) The material has superior catalytic performance; iii) The material should be placed in the position with the right water flow, to provide the sufficient driving force for the piezoelectric catalytic reaction. In addition, if piezoelectric material is added to the shell of a ship or underwater facility, the degradation of organic pollutants and even electricity for the ship or equipment can be achieved at the same time. Likewise, the degradation of pollutants in groundwater and wastewater also can be achieved by piezoelectric materials. Developing piezoelectric catalytic technology that can harness natural driving forces will pave the way for its practical application in the environment.

(III) Current research on piezoelectric catalytic degradation focuses on pollutants in a single laboratory environment, neglecting the complexity of actual environmental conditions. Therefore, more research on piezoelectric catalytic degradation of pollutants should be carried out in open environments such as lakes, rivers, and seawater.

(IV) Although most research related to piezoelectric catalysis describes the piezoelectric mechanism, more evidence is needed to clarify whether mechanically excited charges in the material or intrinsic free charges are involved in piezoelectric catalysis. It is well-known that the internal electric field formed in piezoelectric materials can enhance the efficiency of photocarrier migration, thereby improving the photocatalytic effect. However, no quantitative relationship has been found between the internal electric field and the photogenerated electrons. Therefore, more advanced characterization techniques and in-situ characterization techniques are needed to quantify this relationship for more efficient catalytic performance, such as electron paramagnetic resonance, X-ray photoelectron spectroscopy, and atomic force microscope.

## CRediT authorship contribution statement

Bo Liu: Writing - original draft, Data curation, Conceptualization. Xiaolu Liu: Writing - original draft, Data curation, Conceptualization. Yang Li: Investigation. Muliang Xiao: Investigation. Zhongshan Chen: Investigation. Suhua Wang: Validation. Hongqing Wang: Writing-review & editing. Xiangke Wang: Writing-review & editing, Supervision.

## Declaration of competing interests

The authors declare no conflicts of interests.
