# Supplementary material for: Piezocatalytic techniques and materials for degradation of organic pollutants from aqueous solution
Source: Eco Environ Health. 2024 Aug 22;3(4):418–24. doi: 10.1016/j.eehl.2024.08.001 (PMC11567119; doi:10.1016/j.eehl.2024.08.001)
Supplement: Multimedia component 1 [file mmc1.docx]

**Supplementary Information**

**Piezocatalytic techniques and materials** **for** **degradation of organic pollutants from aqueous solution**

Bo Liu^a,b,1^, Xiaolu Liu^b,1^, Yang Li^b^, Muliang Xiao^b^, Zhongshan Chen^b^, Suhua Wang^c^, Hongqing Wang^a,^*, Xiangke Wang^b,^*

^a^School of Chemistry and Chemical Engineering, University of South China, Hengyang 421001, China

^b^College of Environmental Science and Engineering, North China Electric Power University, Beijing 102206, China

^c^School of Environmental Science and Engineering, Guangdong University of Petrochemical Technology, Maoming 525000, China

*****Corresponding authors. Email: [hqwang2001cn@126.com](mailto:hqwang2001cn@126.com) (H. Wang); [xkwang@ncepu.edu.cn](mailto:xkwang@ncepu.edu.cn) (X. Wang)

^1^ These authors contributed equally to this work.

**
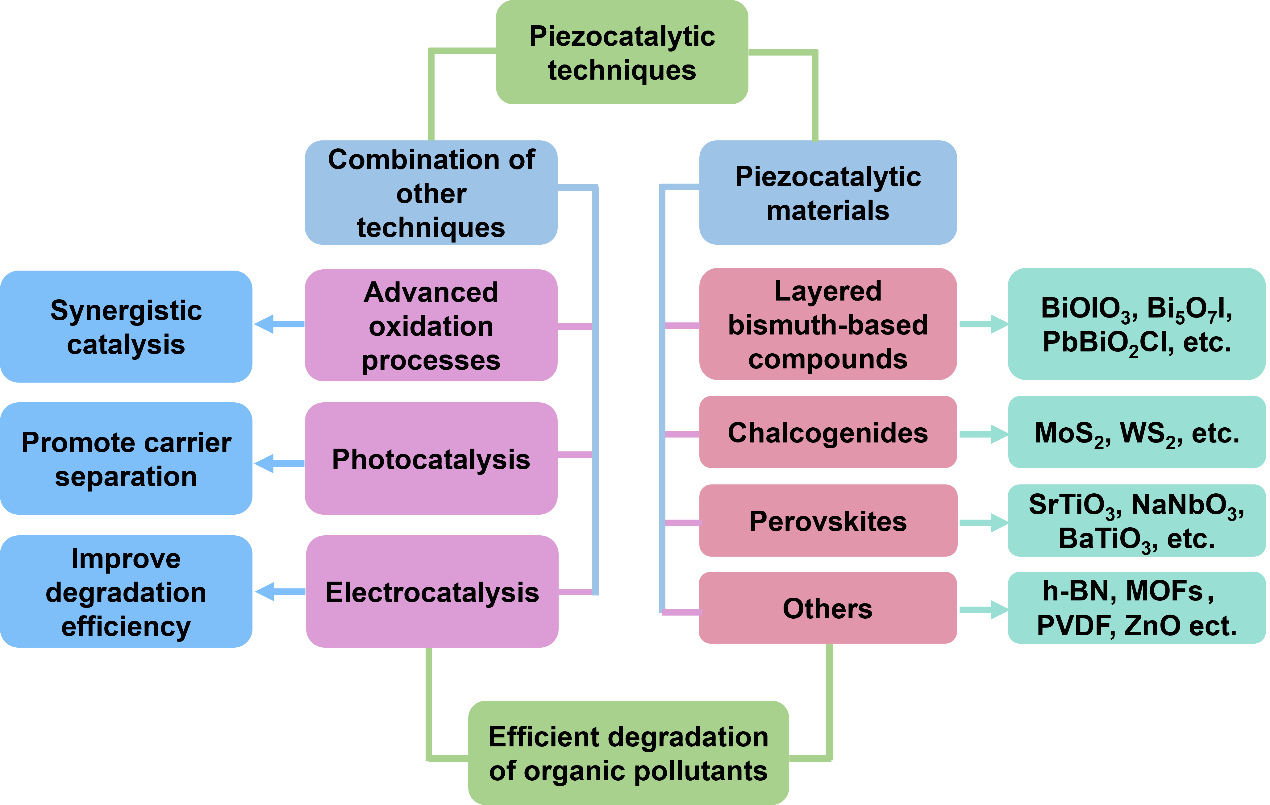
**

**Fig. S1** Piezocatalytic techniques and materials for application of degradation of organic pollutants from aqueous solution.


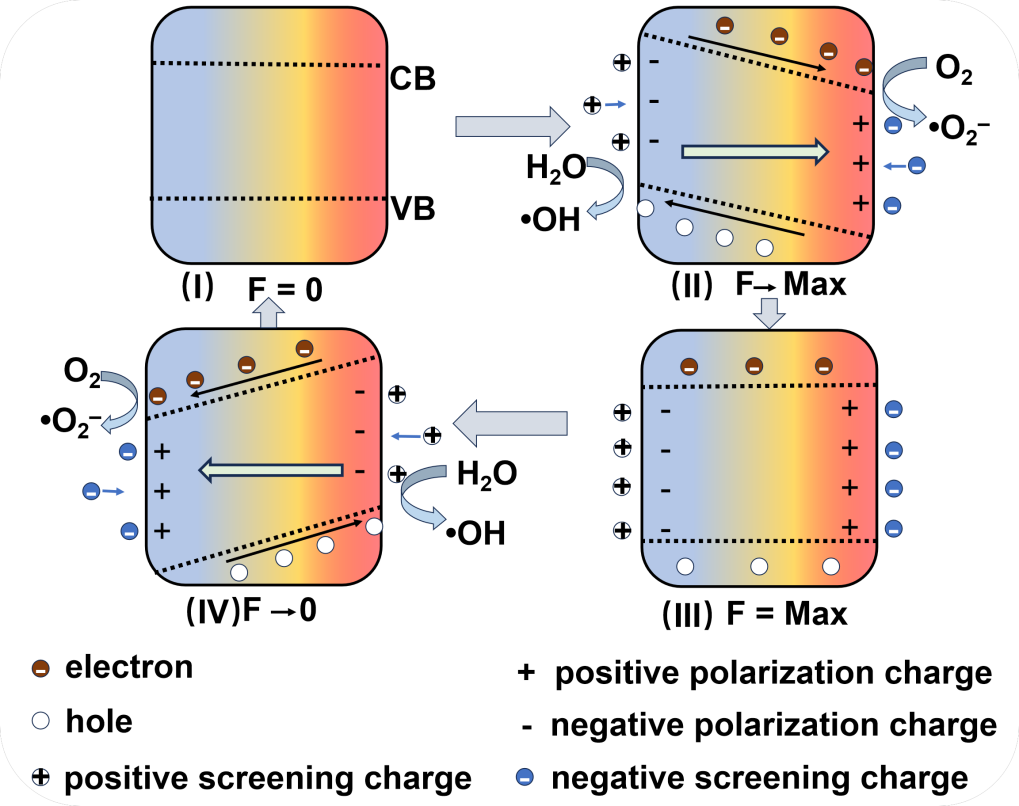


**Fig. S2** Schematic diagram of charge transfer mechanism in piezoelectric catalysis. (I) In the absence of external mechanical force applied to piezoelectric material, the electronic states that are occupied maintain equilibrium, while the surface of the material is uncharged. (II) When mechanical vibration is applied to piezoelectric materials, their internal crystal structure undergoes deformation, causing the separation of positive and negative charge centers, thus generating a piezopotential that can change the electronic energy levels of CB and VB further within the piezoelectric material to promote e^-^ and h^+^ migration. (III) Charges excited by external force or inherent free charges in the piezocatalytic material are efficiently separated and subsequently propelled towards the material's surface driven by the piezopotential. (IV) When the accumulated charges on the surface of the material neutralize the internal electric field, the system returns to an equilibrium state.

| **Table S1** The advantages and disadvantages of different modification strategies. | | |
| --- | --- | --- |
| Modification strategies | Advantages | Disadvantages |
| Morphology modulation | Morphological modulation enables targeted control of material structure and properties, such as large specific surface area, abundant catalytic sites, shorter electron transport distance and enhanced piezoelectric response. | It is difficult to prepare controllable micromorphology and greatly improve catalytic performance. |
| Oxygen vacancy | Oxygen vacancy can increase active sites and act as traps to inhibit charge carrier recombination and prolong carrier lifetime, thereby regulating the electronic structure, promoting more electron hole pair separation and increasing carrier density. | Oxygen vacancy can also become a charge carrier recombination center and affect dipole alignment. |
| Elemental doping | Elemental doping can increase active sites, enlarge the lattice asymmetry and adjust energy band structure to promote the separation and migration of piezoelectric charges, thereby improving the piezoelectric response and reducing the reaction barrier. | It has negative effects if the correct ions are not selected, and the appropriate doping amount is not controlled. During the doping process, there are some problems such as lack of uniformity, high cost of precious metal doping, difficulty in precise structure control and poor cycling performance. |
| Polarization enhancement | Enhancing polarization can increase the crystal dipole moment and asymmetry to improve the piezocatalytic performance. | The polarized charges may cause a shielding effect. |
| Heterojunction construction | The construction of heterojunction is conducive to the rearrangement of interfacial electrons and the separation and transfer of charge carriers, thus reducing the energy barrier of chemical reactions and promoting catalytic reactions. | The electron transfer path may not be as expected, and there are challenges for material collocation, rational design and precise synthesis control. |
| Polymer composites | Polymer composites can make up for the deficiencies of a single material, and the required structure and properties can be customized by selecting the right material and synthesis method. It generally has the characteristics of strong flexibility and no pollution. | The quality is not easy to control, and the material may easily deform and have poor reversibility. |
